# Role of CMR Imaging in Diagnostics and Evaluation of Cardiac Involvement in Muscle Dystrophies

**DOI:** 10.1007/s11897-021-00521-2

**Published:** 2021-07-28

**Authors:** Edyta Blaszczyk, Jan Gröschel, Jeanette Schulz-Menger

**Affiliations:** 1 Working Group on Cardiovascular Magnetic Resonance, Experimental and Clinical Research Center a joint cooperation between the Charité – Universitätsmedizin Berlin, Department of Internal Medicine and Cardiology and the Max-Delbrueck Center for Molecular Medicine, and HELIOS Klinikum Berlin Buch, Department of Cardiology and Nephrology, Berlin, Germany; 2 DZHK (German Centre for Cardiovascular Research), Berlin, Germany

**Keywords:** Muscular dystrophy, Cardiovascular magnetic resonance, Cardiac involvement, Heart failure, Fat, Fibrosis

## Abstract

**Purpose of Review:**

This review aims to outline the utility of cardiac magnetic resonance (CMR) in patients with different types of muscular dystrophies for the assessment of myocardial involvement, risk stratification and in guiding therapeutic decisions.

**Recent Findings:**

In patients suffering from muscular dystrophies (MD), even mild initial dysfunction may lead to severe heart failure over a time course of years. CMR plays an increasing role in the diagnosis and clinical care of these patients, mostly due to its unique capability to precisely characterize subclinical and progressive changes in cardiac geometry, function in order to differentiate myocardial injury it allows the identification of inflammation, focal and diffuse fibrosis as well as fatty infiltration. CMR may provide additional information in addition to the physical examination, laboratory tests, ECG, and echocardiography.

**Summary:**

Further trials are needed to investigate the potential impact of CMR on the therapeutic decision-making as well as the assessment of long-term prognosis in different forms of muscular dystrophies. In addition to the basic cardiovascular evaluation, CMR can provide a robust, non-invasive technique for the evaluation of subclinical myocardial tissue injury like fat infiltration and focal and diffuse fibrosis. Furthermore, CMR has a unique capability to detect the progression of myocardial tissue damage in patients with a preserved systolic function.

## Introduction

Muscular dystrophies (MDs) are a heterogeneous group comprising progressive myopathic disorders. The severity, age of onset, rate of progression, and prognosis vary greatly between the different forms. Duchenne muscular dystrophy (DMD) is the most common inherited muscular disorder of childhood, while myotonic dystrophy (DM) is the most common form in adults followed by facioscapulohumeral muscular dystrophy (FSHD), limb girdle muscular dystrophies (LGMDs), Emery-Dreifuss muscular dystrophy (EDMD), and other less frequent forms [1]. Table 1 provides an overview of the pathologies discussed in this review.

**Table 1:** Overview of muscular dystrophies and the associated cardiac abnormalities

	General			Cardiologic manifestations				
				Clinical			CMR	
Disease	Pathology	Inheritance	Age of onset of primary manifestation	Cardiac involvement (%)	Potential phenotype	Common/typical conduction disturbances/arrhythmias	LGE	Fat/Water
Duchenne muscular dystrophy	Dystrophinopathy	X-linked recessive	3–5 years	Up to 90 [2]	DCM	Sinus tachycardia, ventricular tachycardias	+	+
Becker muscular dystrophy	Dystrophinopathy	X-linked recessive	6–20 (variable)	60–70 [3]	DCM	AV nodal and bundle branch blocks	+	No data
Emery-Dreifuss muscular dystrophy	Nuclear envelopathies, laminopathies	X-linked recessive, autosomal dominant or recessive	10–20 years	50–90 (highly variable) [4]	DCM, HCM, LVNC, biatrial dilation	Bradycardias (AV blocks), tachycardias (SVTs)	Rare, if present associated with tachycardias	Rare
Limb girdle muscular dystrophy	Calpainopathies, sarcoglycanopathies, etc.	Autosomal dominant or recessive	5-20	25–90 [5]	DCM, HCM	SVTs, VTs	+	+
Myofibrillar myopathy	Desminopathy, etc.	Autosomal dominant (other forms exist)	2–50 (highly variable)	15–30 [6]	DCM, HCM, LVNC	Complete AV block	No data	No data
Facioscapulohumeral muscular dystrophy	DUX/SMCHD1 gene	Autosomal dominant (other forms exist)	0–20	5–15 [7]	DCM (rare)	RBBB, SVTs	+	+
Myotonic dystrophy type 1	DMPK gene	Autosomal dominant	0–50 (highly variable)	60–80 [8]	DCM, HCM	AV Blocks, QTc/QRS prolongation, VES, Afib, Aflatter, RBBB/LBBB, VTs	+	No data
Myotonic dystrophy type 2	ZNF-9 gene	Autosomal dominant	30–50	Up to 25 [9]	DCM, HCM	Afib	+	+

Despite the great variety, all of them have three features in common: 1) they affect the musculoskeletal apparatus leading to weakness and progressive disabilities; 2) they typically affect patients at a young age from 5 to 30 years; 3) they all can present with cardiac involvement manifested as heart failure, atrial and/or ventricular arrhythmias, and conduction abnormalities. It is important to verify whether the symptoms, e.g., dyspnea, fatigue, chest pain, are caused by cardiac disorders. Especially, the progressive muscle weakness and the significantly reduced mobility can mask cardiac involvement. Arrhythmias and conduction abnormalities can often be symptomless even in advanced stages of the disease. Therefore, it is important to assess whether a subclinical cardiac involvement is present.

In some MDs, such as DMD and BMD, cardiac involvement is well known and may occur in up to 90% of patients [2]. Due to the very high risk of developing a dilated cardiomyopathy (DCM), these patients should receive prophylactic heart failure treatment even before the onset of symptoms [10]. That is the reason why, especially in DMD and BMD patients, it is important to identify cardiac involvement as soon as possible, in order to initiate a possibly cardio-protective therapy and to limit the progression of heart failure [11].

In the recent years, progress was made regarding the treatment of patients with MD. Available therapies can help to reduce symptoms. Ongoing research on new treatment strategies including gene-based approaches are promising in different types of MD. Most of them are focusing on neuromuscular limitations with the intention to preserve the functional capacity as long as possible. Cardiovascular manifestations are targeted as well in studies, and first results confirmed that a proactive strategy of early cardiological diagnosis and treatment is essential to maximize the duration and quality of life of these patients [10, 12, 13]. The results of these studies are reflected in present recommendations for diagnosis and management of DMD patients advocating a complete cardiac evaluation and early initiation of cardio-protective therapies [14]. Following the latest DMD recommendations, cardiovascular magnetic resonance (CMR) should be considered for baseline assessment in DMD patients already at the age of 10, when it can typically be done without anesthesia [15••].

CMR is accepted as the reference method for assessing ventricular and atrial volumes, systolic function, and regional wall motion abnormalities. CMR is a non-invasive imaging technique that has the unique capability to detect and follow the progression of myocardial tissue damage such as edema, focal and diffuse fibrosis, and fat infiltration, even in the context of a preserved systolic function. Because of its high spatial resolution, CMR can differentiate various scar patterns and detects areas with diffuse fibrosis or edema, e.g., with T1- and T2-mapping techniques. There is growing evidence underlying the strength and additive value of CMR in identifying patients at risk in different cardiac diseases. [16] The presence of focal fibrosis in patients with non-ischemic cardiomyopathies strongly predicts adverse cardiac outcome and is a negative predictor of functional recovery and prognosis [17–19]. The presence of focal fibrosis in patients with hypertrophic cardiomyopathy, a so-called risk modifier, helps in the decision-making for borderline patients regarding an implantable cardiac defibrillator (ICD). Furthermore, diffuse myocardial fibrotic changes seem to be independently associated with complex ventricular arrhythmias [20], and an identification of myocardial edema is associated with a long-term outcome [21]. Similarly, DMD and BMD are often associated with myocardial fibrosis as a potential substrate for ventricular arrhythmias which can increase in burden load concurrently with worsening of myocardial remodeling and function [16, 22, 23].

Nowadays, with increasing life expectancies of patients, attending physicians have to take lifestyle choices, comorbidities, and diseases of the elderly into consideration when approaching the individual patient and therapy. Elder patients with muscular dystrophies prove to be a challenge as they often exhibit age-related disorders like atherosclerosis, metabolic impairments in the form of elevated lipid levels or diabetes mellitus, wear-and-tear diseases of the musculoskeletal system, as well as neurocognitive disorders, which can mimic and exacerbate symptoms of the underlying muscular conditions. That differs depending on age and the kind of MD. Most of the patients are young and an exclusion of non-ischemic myocardial injury is usually the first-line approach. Since markers of cardiac injury, like troponin or CK-MB, are very often elevated, the confirmation or exclusion of active inflammation and detection of fibrosis applying CMR is most important and protocols fitted for the detection of myocarditis are usually the first-choice. Of course, in cases where patients present symptoms of CAD and co-existing risk factors, we use CMR stress-protocols for a fast and accurate detection of hemodynamically relevant coronary artery stenoses.

This review will give an overview of the current recommendations, new evidence, and how these patients should be managed in a team effort of neurologists and cardiologists. Regarding the cardiac involvement, we will focus on CMR findings and its diagnostic role in the detection of myocardial involvement, risk stratification, guiding therapeutic management, and follow-up in patients suffering from MD.

## Duchenne and Becker Muscular Dystrophies

DMD and BMD are classified as dystrophinopathies as both are due to X-linked recessively inherited mutations in the gene that encodes for the protein dystrophin. Major differences between the pathologies are the type of mutations, onset, severity, and progress of symptoms. The majority of DMD will develop a cardiomyopathy by the age of 18. The available date shows a median survival of 24 years for a cohort of patients with molecularly confirmed DMD. In BMD patients, the frequency of cardiac involvement can reach up to 60–75%. The average life expectancy in this patient group is about 40 to 50 years and death is most commonly due to dilated cardiomyopathy [24].

DMD, with an estimated prevalence of 1 per 3000 births, the most common hereditary muscular dystrophy, usually starts showing signs at an age of 3–5 years with a severe and progressive clinical course. The proximal lower limb muscles are affected first leading to a waddling gait, inability to jump or run, the pathognomonic Gowers sign, and wheelchair dependency by the age of 12. During this non-ambulatory phase, a progressive decline in ventilatory capacities sets in leading finally to the need for non-invasive ventilation. Majority of patients do not survive their third decade of life due to respiratory or cardiac failure [2]. DCM and impaired left ventricle systolic function are the primary causes of cardiac death in a high proportion of patients with DMD and BMD. The frequency of cardiac involvement can reach up to 90% [25]. Interestingly, Winterholler et al. could identify a connection between the increased risk for ischemic strokes and DMD-associated cardiomyopathy without evidence of atrial fibrillation. DCM was the only risk factor for ischemic stroke in all patients [26].

**Fig. 1:**
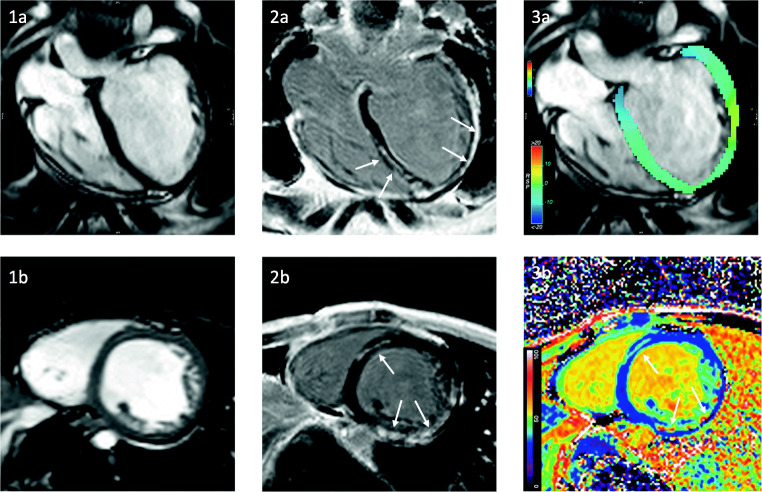
LV dilatation (EDV 201 ml) and severe systolic dysfunction (LVEF 32%) in a 21-year-old patient with DMD. Cine imaging in a long-axis four-chamber view (**1a**) and short axis (**1b**) and reduced global longitudinal strain (− 10.6%) (**3a**). Late Gadolinium Enhancement showing extensive focal non-ischemic enhancement in LV (arrows, **2a-b**) corresponding with increased extracellular volume (ECV) in short axis (arrows **3b**)

Persistent sinus tachycardia is the most common detectable rhythm deviation from normal sinus rhythm in DMD. In addition to the DCM, patients can present with signs and symptoms of ventricular and supraventricular tachycardias [27–29], mitral and tricuspid insufficiency, left-sided heart failure, and SCD [30]. Current expert care considerations have advised to establish baseline evaluation by resting ECG and a non-invasive imaging method, either transthoracic echocardiography (TTE) or CMR with an annual follow-up [15••]. CMR is the preferred imaging modality for baseline assessment in patients with poor acoustic window offering, in addition to other non-invasive imaging methods, the ability to detect macroscopic findings like fat infiltration or focal fibrosis by late gadolinium enhancement (LGE) [31, 32] (Fig. 1). However especially in younger patients until the age of 6–7 years, TTE is the preferred imaging method due to a lower compliance [15••]. The myocardial fibrosis in DMD has a progressive course and correlates with left ventricular (LV) dilation and reduction in the ejection fraction (EF) [33, 34]. The presence of myocardial fibrosis detected by LGE has negative implications on the prognosis and is associated with an increased risk for cardiovascular adverse events [13]. Even in the absence of LGE, CMR seems to be able to detect myocardial damage by the application of parametric mapping techniques. These novel methods play an increasing role in CMR, especially regarding the evaluation of inflammation and diffuse, subclinical fibrotic changes. Patients with DMD often have increased native T1 and extracellular volume (ECV) values in myocardial regions without LGE, and therefore mapping techniques seem to have the unique ability to identify early cardiac changes in DMD [35]. Interestingly, a recent study showed an inverse relationship between LGE-positive segments and T1 values attributing this to a fibrofatty replacement [36]. Further research is needed to verify the aspect of fat infiltration as a major contributor to the pathophysiology. Furthermore, DMD patients with myocardial inflammation in CMR show a rigorous progression to heart failure [37, 38].

As far as medical treatment goes, the focus should lie on an early initiation of heart failure treatment if possible when abnormalities are found during imaging even before the onset of symptoms [15••]. Current standard of care should follow adult heart failure guidelines by starting with angiotensin-converting enzyme inhibitors (ACE) or angiotensin II receptor blockers (ARB) [14, 39]. Recent evidence suggests that the mineralocorticoid antagonists (MRA) spironolactone and eplerenone could possibly delay the decline of LV contractile function in DMD patients [40, 41]. So far, the use of β-adrenergic blockade (beta blockers (BB)) is recommended only in patients who present with a reduced EF at baseline. Without other indications, e.g., arrhythmias, the prescription of BB to delay or prevent the onset of DCM is currently not recommended. Patients with fluid retention due to ventricular dysfunction should be treated with diuretic agents. Currently, there is no data to support the routine use of glucocorticoids (GCS) in order to improve cardiac function; however, GCS might be prescribed to slow the progression of cardiac disease in patients with DMD [42].

Female carriers of DMD or BMD are also at risk of developing a cardiomyopathy. In contrast to DMD males, female carriers may not develop clinically apparent peripheral muscular disease but can instead present with a wide range of cardiac disorders including heart failure and SCD [43]. Cardiac involvement may develop in up to 50% of cases [44].

**Fig. 2:**
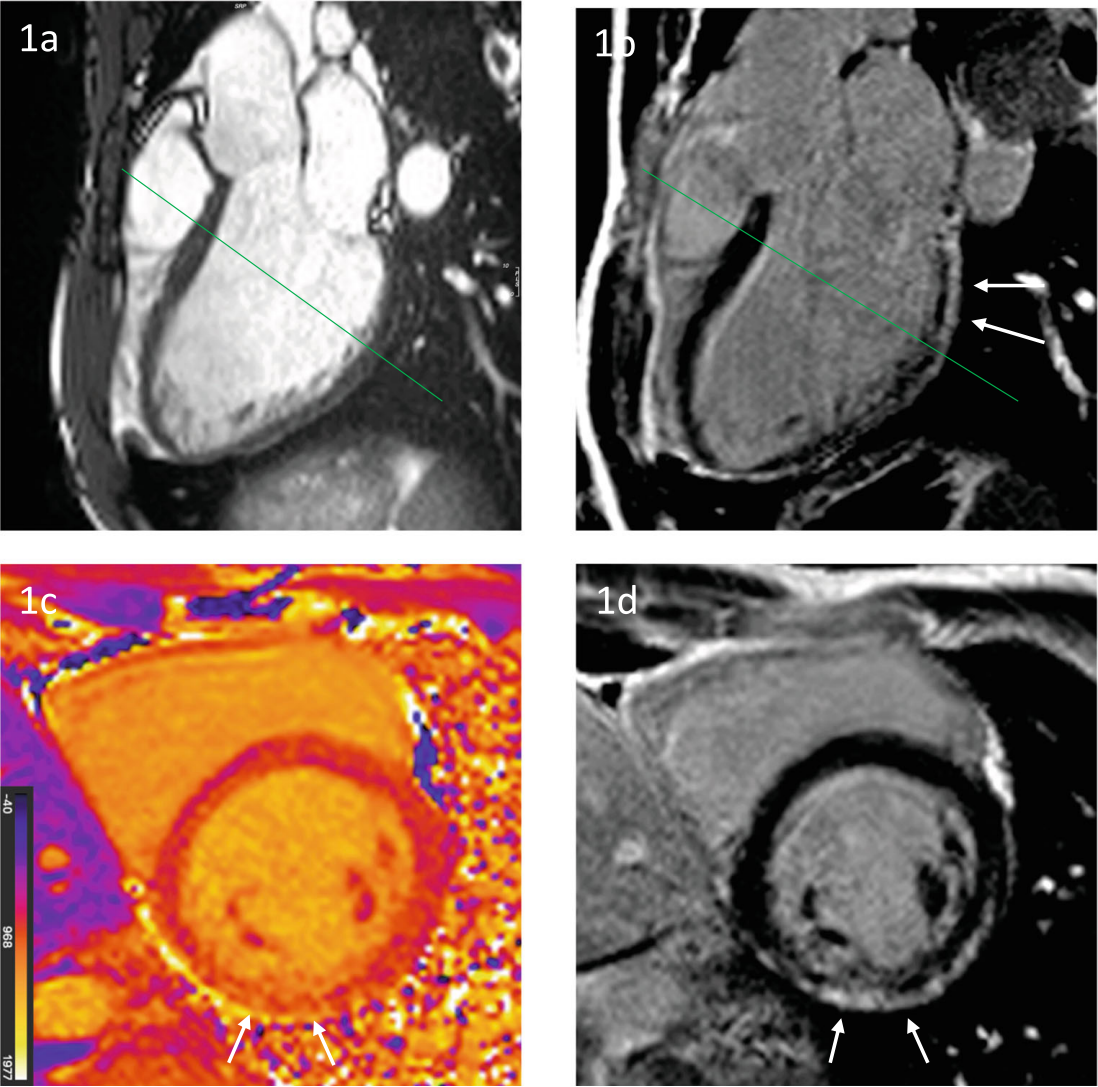
LV dilatation (EDV 205 ml) and mild systolic dysfunction (LVEF 49%) in a 27-year-old patient with BMD. Cine imaging in a long-axis three-chamber view (**1a**) and corresponding image in late gadolinium enhancement with focal non-ischemic enhancement within inferolateral wall in long (arrows, **1b**) and short axes (arrows **1d**). Native T1 map with increased T1 native values corresponding to LGE in the short axis (arrows **1c**). Green lines: slice location of the short axis

BMD, incidence 1:18,000, on the other hand, is the milder form of the dystrophinopathies. Onset of symptoms starts usually between the 6th and 20th year of life and patients keep their ability to walk until the age of 16 or sometimes even longer. Interestingly, cardiac involvement is often more prominent than musculoskeletal symptoms and the major contributor to the symptomatic load [3, 45]. Approximately 70% of BMD patients develop a DCM, mostly in the third decade of life or later. Usually, the degree of skeletal muscle involvement does not correlate with the severity of the cardiomyopathy. Furthermore, cardiac death seems to be more common in BMD than in DMD [24]. Early echocardiographic studies in the 1990s could already show that especially right ventricular wall motion abnormalities are observable [24]. Twenty years later, Yilmaz et al. used CMR in their cohort and could not only confirm these results but on top prove that LGE goes hand-in-hand with a reduction of LV ejection fraction (LVEF) [23]. Typically, LGE in BMD follows a subepicardial or midwall pattern with progression to a transmural scar and is located in the inferolateral region of the LV (Fig. 2). Our approach to cardiovascular care and treatment is similar to the one outlined above for DMD especially in the light of recent evidence that even very young patients in their 2nd decade of life could have already first signs of myocardial fibrosis [46]. Complete cardiac evaluation should begin at approximately the age of 10 or at the onset of signs and symptoms. Cardiological evaluations should continue at least biannually [44].

## Emery-Dreifuss Muscular Dystrophy

**Fig. 3:**
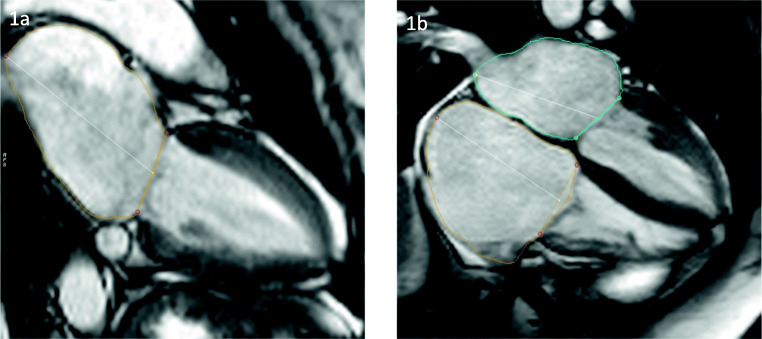
Biatrial dilatation in a 43-year-old patient with EDMD. Cine imaging in a long-axis two- (**1a**) and four-chamber view (**2a**). Right atrium 45 cm2. Left atrium 30 cm2, 89 ml/m2

In contrast to the aforementioned muscular dystrophies, EDMD, also called humeroperoneal muscular dystrophy, can be inherited as an X-linked recessive, autosomal recessive (AR), or autosomal dominant (AD) disease, the latter one being the most common. The main reason for this heterogeneity is the wide array of mutations accounting for the disease. So far 7 subpopulations (EDMD1-EDMD7) have been identified with the majority having variants in genes encoding nuclear membrane proteins, therefore termed nuclear envelopathies. The AD and AR forms, for example, can be linked to the LMNA gene encoding for lamin A and C (EDMD2 and EDMD3) as well as to the SYNE1 and SYNE2 genes encoding nespirin 1 (EDMD4) and 2 (EDMD5), respectively [47]. The X-linked forms, EDMD1 and EDMD6, on the other hand, are due to mutations on the EMD gene encoding for emerin and the FHL1 gene encoding for LIM domain protein 1, respectively. As more and more genes and mutations are deciphered, the link between muscular and cardiac abnormalities becomes clearer. Recent research suggests that patients with mutations in the EMD gene can present solely as a cardiac phenotype, termed cardiac emerinopathy, with probable association with left ventricular noncompaction (LVNC) and an increased risk of thromboembolic events [48]. The classic triad consists of contractures, especially of the elbow, humeroperoneal muscle weakness, and cardiac disorders. The cardiac manifestations include DCM and associated symptoms, SCD, and atrioventricular rhythm abnormalities [49, 50]. Cardiac involvement in EDMD often appears as atrial dilatation in different stages (Fig. 3) which could explain the common occurrence of supraventricular tachycardias, atrial fibrillation, and atrial flutter in very young patients [51, 52]. Rhythm disturbances can also be of bradycardic nature (atrioventricular blocks, total atrial standstill) [52]. Macroscopic LGE changes are not very common; however, in some cases with SCD due to a ventricular tachycardia, fibrotic remodeling with fat deposits in the area of ​​the conduction system was reported [53, 54]. Current guidelines of the European Society of Cardiology (ESC) recommend implantation of a pacemaker (PM) as soon as first bradyarrhythmias or other conduction abnormalities appear before the 30th year of life [55]. As Russo and Nigro [56] mention in their letter to the editors, there is evidence that ventricular events in EDMD can be prevented by proper ICD implantation, and therefore recommend the combined implantation of PM-ICD devices in patients with preserved LVEF. Given the frequent implantation of PM-ICDs [57] and the rarity of the disorder, estimated at 1:100.000, CMR studies are limited to case series [58].

In addition to annual monitoring for arrhythmias with 24h-ECG and evaluation of dilatation progress with TTE, we follow the above-mentioned guidelines. Because SCD can be the first presentation of cardiac involvement in this patient group, cardiac screening of individuals with EDMD and first-degree relatives (including female carriers of X-linked EDMD) is recommended [59].

## Limb Girdle Muscular Dystrophy

LGMDs are a very heterogeneous group of disorders divided into an autosomal dominant form LGMD1 and an autosomal recessive form LGMD2. Additionally, each group has several subclassifications (e.g., LGMD1A-LGMD1H) based on the gene and the muscular protein involved. In 2018 during the European neuromuscular center (ENMC) international workshop, a new classification system was proposed to better replicate the new insights into the pathogenesis of LGMD [60]. As a complete description would go beyond the scope of this review, we will focus on the forms most commonly associated with cardiac manifestations (in brackets the former name, gene, and the corresponding protein): LGMDR4 (LGMD2E, SGCB, beta-sarcoglycan) and LGMDR9 (LGMD2I, FKRP, fukutin-related protein). Two additional pathologies, in the old classification termed LGMD1B and LGMD1E, have been excluded and reclassified under different names with the first one being attributed to the EDMD group and the latter now named myofibrillar myopathy (MFM). Around 60% of patients with LGMDR4 have cardiac manifestation including DCM, hypertrophic cardiomyopathy (HCM), conduction abnormalities, and increased risk of SCD [61]. Similar changes can be observed in LGMDR9 where the decline in LVEF correlates with mortality [62]. The levels of creatinine kinase (CK) are usually severely elevated and are one of the most common findings on presentation [63].

**Fig. 4:**
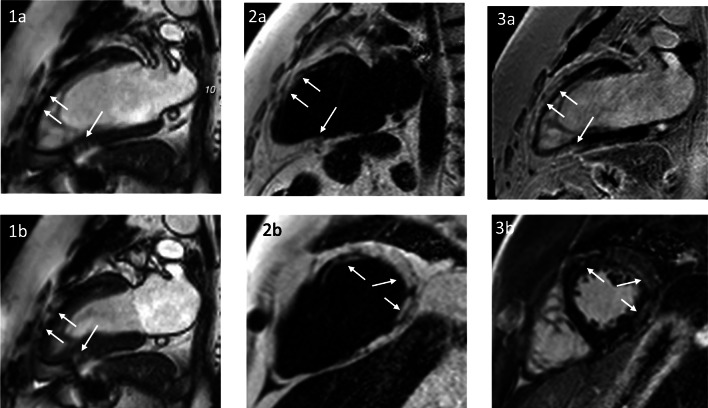
A 45-year-old patient with LGMD2I and normal LV function (LVEF 57%). SSFP cine in a two-chamber view in end-diastole (**1a**) and end-systole (**1b**) with no wall motion abnormality, but with detectable myocardial structure abnormality (arrows). A 2-chamber view and a midventricular short axis. Fat/water imaging showing extensive epicardial fat with subepicardial and intramural fatty replacement of the myocardium (arrows **2a**, **b**). Fibrosis imaging (LGE) showing a bright signal—indicating a scar, but the bright signal indicates fatty replacement as well (arrows **3a**, **b**). Only the combination of LGE and fat imaging allows the differentiation of the tissue character

Possible findings during CMR exams include dilation of the ventricles with diastolic dysfunction, subclinical fibrosis, and increased cardiac fat tissue [64, 65]. Myocardial fat infiltration is less studied, but due to recent technical developments, the identification of fatty changes has become significantly easier (Fig. 4). Cardiac care should follow similar principles as above, with annual visits to exclude progress of arrhythmic episodes. Variants that are not associated with an increased cardiac risk can be followed every 2 years.

## Myofibrillar Myopathy

Formerly classified under LGMDs, this disease now entails its own clinical spectrum. The genetic mechanism for the disorder has been mapped to genes involved in desmin metabolism leading to accumulation of faulty protein aggregates. Especially, the mutation in the DES gene is associated with cardiac symptoms [66]. Cardiomyopathies associated with MFM can present very heterogeneously, ranging from dilated to hypertrophic to restrictive, or even LVNC phenotypes [42]. Cardiac care should emphasize the detection of conduction abnormalities [42, 67]. The decision regarding the implantation of a prophylactic ICD in this patient group should not depend on EF alone but rather on the synopsis of all findings and evaluations [42].

## Facioscapulohumeral Muscular Dystrophy

FSHD is the third most common inherited muscular dystrophy, behind DMD and the myotonic dystrophies. Inheritance pattern is mostly AD with frequent variants due to sporadic mutations. Most cases, 95%, can be mapped to the DUX4 gene on chromosome 4. Deletion of repeat units on the long arm on the chromosome leads to the subtype FSHD1. In contrast to this, FSHD2, although phenotypically resembling FHD1, is due to mutations of the SMCHD1 gene. As the group name indicates, the muscle weakness is usually located in the facial, scapular, humeral, and abdominal region, often with an asymmetric involvement. Age of onset is highly variable, ranging from infancy to the age of 20 [68]. In comparison to other muscular dystrophies, cardiac involvement is less common and presents in the majority of cases with conduction abnormalities, especially incomplete right bundle branch blocks [7] and supraventricular arrhythmias [69]. Few case reports and series describe the DCMs and SCDs in patients with FSHD but there is a lack of larger prospective studies. In a recent CMR study, we could show that patients with FSHD can have myocardial tissue changes despite a normal systolic function. In our cohort, we found LGE in 25% of patients, most commonly in the basal inferolateral, inferior, and septal segments. Native T1 and ECV values were significantly higher not only in LGE-positive segments but also in the adjacent regions, suggesting a diffuse interstitial fibrosis. Areas of fatty infiltration were found in 13% of patients [70]. Nikhanj et al. showed recently that there is evidence of elevated biomarkers, e.g., high sensitive troponin, with clinical relevance for the diagnosis of cardiomyopathies and prognosis of cardiac adverse events in patients with MD [71]. As this new emerging evidence for cardiac disease in FSHD has not been incorporated into guidelines yet [72], we would like to explain our approach to these patients. Follow-ups are done annually with TTE and 24h-ECG. If symptoms are progressive or new arrhythmias are detected on 24h-ECG, despite stable echocardiographic findings, we perform a control CMR exam. Other cardiac comorbidities, like chronic coronary syndrome, CHF, or arterial hypertension (HTN), are treated according to guidelines [39, 73, 74].

## Myotonic Syndromes

The myotonic syndromes are a group of disorders that share the common trait of delayed muscular relaxation. Interestingly, the two more common disorders, myotonic dystrophy type 1 (DM1) and myotonic dystrophy type 2 (DM2), are rather multisystem diseases that can even present with no muscular signs or symptoms at all. The other subgroup, also termed non-dystrophic myotonias, is a group of very rare channelopathies with the primary symptom being muscular dysrelaxation. As they are rare even in the context of all these rather uncommon disorders, we will not describe them here.

DM1, also known as Curschmann-Steinert disease, is an autosomal-dominant disorder due to CTG-repeat-expansions on the DMPK gene. The length of the repeats correlates loosely with the severity of the disease, giving rise to the following phenotypes: congenital, childhood, classic, and mild. The underlying pathophysiology results from the accumulation of faulty RNA that inhibits the transcription of several other genes, a process called RNA toxicity [75]. Examples of genes that are dysfunctional due to transcription are the skeletal muscle chloride channels, the insulin receptor, and cardiac troponin T. This mechanism is one reason for the multiorgan involvement in the DMs, the other one being the somatic mosaicism which results from an inherent instability of the CTG repeats. The instability is especially pronounced in the brain, skeletal muscle, and the heart. Signs and symptoms are presented in Table 2. DM1 is heavily associated with cardiac disease burden, ranging from conduction disorders and arrhythmias to ventricular and atrial structural heart disease with signs and symptoms of overt heart failure [8]. Major abnormalities are AV-blocks, QTc and QRS prolongations, increased ventricular extrasystoles, atrial fibrillation and flatter, right and left bundle branch blocks, as well as non-sustained ventricular tachycardias [76•]. Groh et al investigated the, later so-called, Groh-criteria (rhythm other than sinus, PR >240 ms, QRS >120 ms, or II/III-degree AV/block) in their study and found out that they can be predictors of SCD in patients with DM1 [77]. Very recent studies emphasize that SCD can even be associated in more cases than previously thought, with non-cardiac origin, underlying once more the multisystem characteristic of DM1 [78].

**Table 2:** Signs and symptoms of myotonic dystrophy type 1 and 2

Disease	Subgroup	Age	Muscle involvement	Cardiac	Endocrine	Eye	Gastrointestinal
Myotonic dystrophy type 1			Distal (facial, neck, forearm, foot dorsiflexor), myotonias >myalgias	Conduction disturbances (AV blocks, bundle branch blocks), arrythmias (QT/QRS prolongation, VES, VTs, Afib, Aflatter), DCM, HCM	Hypogonadism, glucose intolerance, hypothyroidism	Cataracts (late in the disease)	Irritable bowel syndrome, gall stones, dysphagia
	Congenital	Birth					
	Childhood	1–11					
	Classical	12–50					
	Mild	>50					
Myotonic dystrophy type 2		30–50	Proximal (hip, neck, elbow, fingers), myalgias >myotonias	Atrial fibrillation, DCM, HCM (less common than in DM1)	Hypogonadism, glucose intolerance, hyperhidrosis	Cataracts (common)	Less common than in DM1

Regarding CMR, DM1 is associated with a non-ischemic pattern of LGE enhancement and ECV even in subclinical stages, indicating that the remodeling process is starting at an early age [79–81]. In a cohort of 80 patients, Hermans et al. found myocardial fibrosis in 13%. It appeared most often as midmyocardial enhancement in the septum and within the basal inferiolateral segments of the LV wall [82]. Recently, it was shown that DM1 patients, who present with an increase in cardiac ECV, often have decreased strain values, which could possibly indicate early cardiac pathology [79]. In addition to annual follow-ups with 24h-ECG and TTE, the focus of cardiologic care should lie on the decision when to implant PM-ICD devices. International guidelines recommend to consider PM placement even in patients with AV-block type I and, at the same time, to assess the need for implantation of an ICD, taking into consideration the future risk of ventricular arrhythmias [83, 84].

**Fig. 5:**
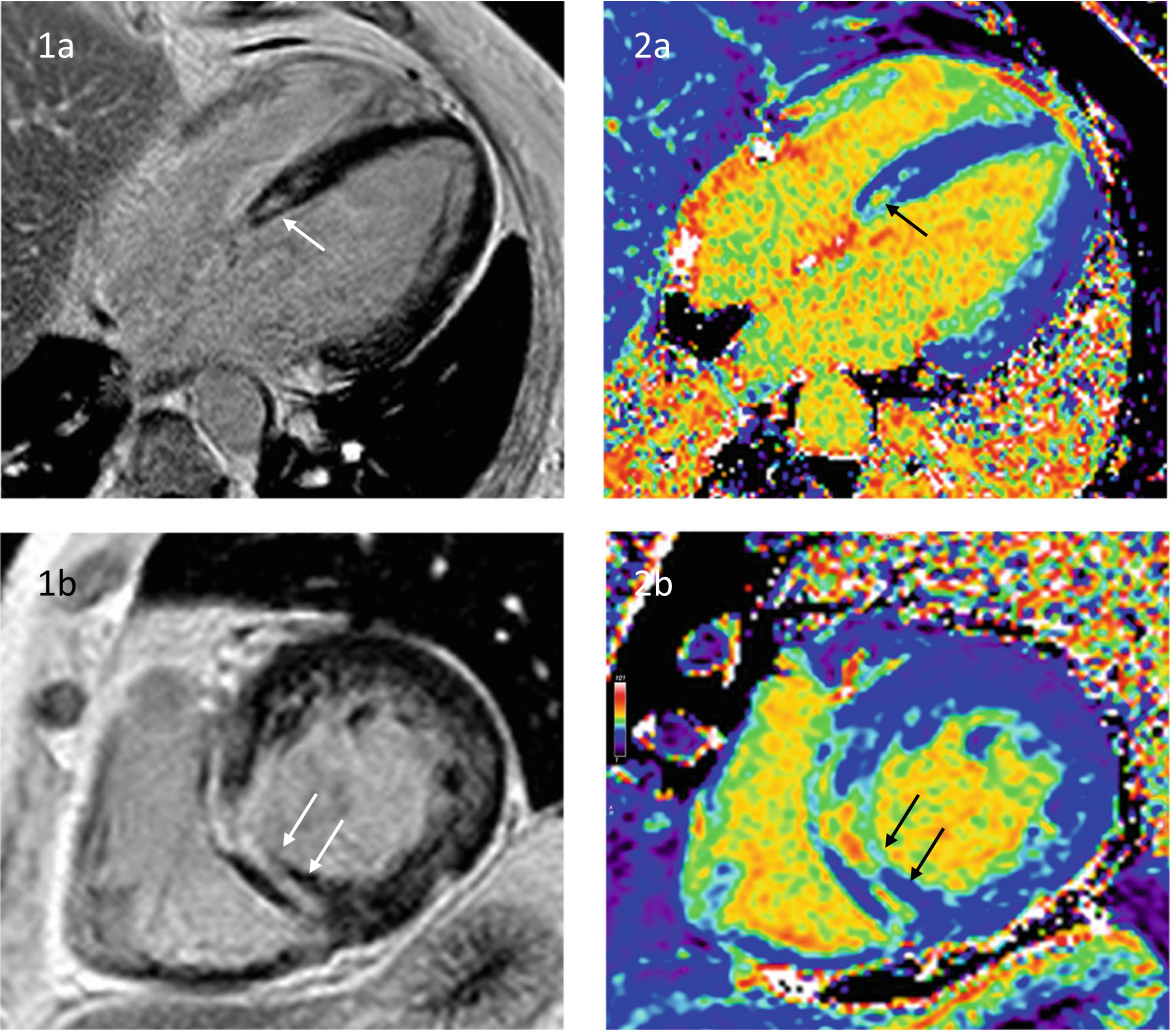
A 57-year-old patient with DM2 and normal LV function (LVEF 58%). Detection of focal non-ischemic enhancement in the LV septum (arrows) in a four-chamber view (**1a**) and short axis (**1b**). Late gadolinium enhancement, corresponding with increased extracellular volume (ECV) in a four-chamber view and short axis accordingly (arrows **2a**, **b**)

DM2 is also transmitted in an AD fashion but instead of a three nucleotide repeat, it is caused by a CCTG tetranucleotide repeat located on the ZNF9 gene. Generally, DM 2 is considered to be a milder form with a later onset, usually in the 30th to 50th decade of life. The characteristic muscular involvement is more proximally orientated; therefore, the disease is also known as proximal myotonic myopathy (PROMM). Interestingly, patients commonly present with myalgias, rather than myotonic symptoms, endocrine disturbances, and presenile cataracts [85] (Table 2). In comparison to DM1, PROMM has always been thought to have less cardiac involvement, but cross-sectional studies suggest that the burden of cardiac involvement is similar and in regards to atrial fibrillation and left ventricular dysfunction even higher in PROMM [9, 86]. The left ventricular dysfunction should be of concern as CMR can already detect myocardial fibrosis and an increase in ECV in subclinical stages with preserved EF (Fig. 5). We could demonstrate that focal fibrosis and fat deposits are detectable in 22% and 21% of patients with DM2, respectively [87]. This opens the possibility to apply an anti-remodeling therapy, e.g., with ACE inhibitors, to holt or even prevent a further decline [88]. Large multicentric and prospective studies are missing on this topic so far. In general, we follow a similar approach as outlined above for DM1. As patients tend to be (a) older when symptoms start and (b) there is a huge variety of symptoms the general practitioner and cardiologist should be suspicious of an underlying genetic abnormality. Care should be taken to properly manage comorbidities like HTN, diabetes mellitus, and hyperlipidemias.

## Conclusion

MDs are rare diseases and therefore no large multicenter studies exist that provide a high level of evidence. But the currently available data indicates that this group of patients deserves special care regarding the early diagnosis of cardiac manifestations and optimal timing of heart failure therapy, including devices if needed. Due to individual predispositions, even mild initial dysfunction may lead to severe heart failure over a time course of years. CMR plays an increasing role in the diagnosis and clinical care of patients suffering from MD. In most of these patients, the LV is either dilated or shows a reduction in systolic function with a reduced EF. However, during the last years, the awareness of a potential impact of the right ventricle (RV) and the atria has increased.

Due to limitations associated with echocardiography such as poor acoustic window and image quality, CMR nowadays plays a key role in screening and management of cardiomyopathies. This especially holds true for MD patients, as their limited capacity to adjust the position for a proper acoustic window reduces image quality even more. Beyond precise assessment of volume and function, CMR has the unique ability to differentiate myocardial tissue including the detection and localization of edema as well as focal and diffuse scars or fibrosis. In our patients, we perform at least one diagnostic CMR scan at baseline to identify the extent of cardiac involvement in order to plan the follow-up examinations and visits.

**Fig. 6:**
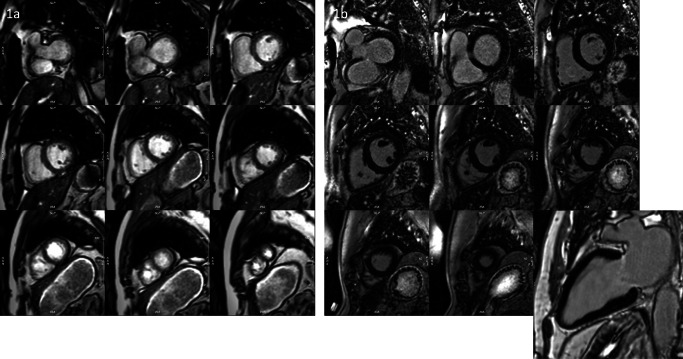
Examples of free-breathing acquisition in a patient with atrial fibrillation using real-time cine stack acquired in 40 s (**1a**) and PSIR MOCO LGE SAX stack acquired in about 3 min (**1b**)

Owing to the development of more robust and faster techniques, scan time has been significantly reduced. Unfortunately, it is still mistakenly believed that CMR is unsuitable for very sick patients even though it can provide unique information to guide further therapy. New technological developments, like real-time and motion-corrected imaging techniques, enable patients with reduced breath-hold capacities or arrhythmias to undergo CMR exams. Furthermore, multi-slice LGE acquisitions are already available as non-breath-hold techniques, reducing scan time by more than 60% (Fig. 6). In follow-up CMR exams, we often apply protocols without contrast media application, solely based on parametric mapping.

CMR has gained increasing clinical relevance especially in recent years leading to class I recommendations in many international cardiological guidelines. Further randomized clinical trials with high level of evidence using CMR as a robust, non-invasive technique are needed to define the impact of early cardiac remodeling and myocardial injury as well as to identify a relation between cardiac involvement and arrhythmias on long-term prognosis and on therapeutic decision-making in patients with different types of MD. In the case of rare diseases, this goal can be achieved by initiating registries that help systematically collect the data from these patients based on CMR findings.
